# Neurolisteriosis: The Importance of a Prompt Diagnosis

**DOI:** 10.7759/cureus.16662

**Published:** 2021-07-27

**Authors:** Clara Silva, Diana Ferrão, Mariana Almeida, Luis Nogueira-Silva, Jorge S Almeida

**Affiliations:** 1 Internal Medicine, Centro Hospitalar Universitário de São João, Porto, PRT; 2 Center for Health Technology and Services Research, Faculty of Medicine of the University of Porto, Porto, PRT; 3 Medicine, Faculty of Medicine of the University of Porto, Porto, PRT

**Keywords:** listeria monocytogenes, immune thrombocytopenia, meningoencephalitis, abscess, immunosuppression

## Abstract

Immune thrombocytopenia (ITP) is a prevalent disease that may need immunosuppressant treatment, which increases the risk of an opportunistic infection. We present the case of a woman with corticosteroid-resistant ITP who was electively admitted to the hospital to initiate second-line treatment. On the second day, she presented with a high fever and altered mental status, with no lesions on the cerebral tomography and inconclusive cerebrospinal fluid analysis. Nonetheless, she was promptly started on empiric antibiotics for meningitis which were then adjusted for *Listeria monocytogenes*, isolated in blood culture. The cerebral magnetic resonance showed signs of cerebritis and pyogenic foci. The patient was discharged after 73 days of treatment, asymptomatic and with no neurological sequelae. The mortality rate of neurolisteriosis can be as high as 50%. The median time between the initial symptoms and their detection is seven days, with many patients already developing cerebral abscesses. The favorable outcome of this patient was due to the precocious detection and start of the treatment.

## Introduction

Immune thrombocytopenia (ITP) is an autoimmune disease, where thrombocytopenia occurs due to autoantibodies against platelet antigens. It can be secondary to other conditions, but it is usually idiopathic. Its treatment is based on immunosuppression, beginning with high-dose corticosteroids, which are later tapered until the minimal effective dosage. In some patients, high doses can be needed for a longer period of time, increasing the risk of opportunistic infections [1].

## Case presentation

We present a case of a 69-year-old Caucasian woman, with type 2 diabetes mellitus, obesity, immune hypothyroidism, and atrial fibrillation, anticoagulated with apixaban. She was diagnosed with ITP (platelet count of 6 x 10^9^/L) and was started on corticosteroids - prednisolone 1 mg/Kg/day. She had a rapid increase in platelet counts and this dose was soon tapered by 10 mg every five days. One month after diagnosis, under prednisolone 5 mg/day, she was admitted to the ER for petechiae throughout the entire body, predominantly in the lower limbs, and fatigue. The blood panel showed a platelet count of 9 x 10^9^/L. She had no other hemorrhagic manifestations, no signs or symptoms of infection, and no other remarkable analytical changes. She underwent methylprednisolone intravenous (IV) pulses of 500 mg/day for three days and then restarted prednisolone 1 mg/Kg/day. She was discharged with a platelet count of 90 x 10^9^/L, maintaining anticoagulation. Two months later, although maintaining a high dose of corticosteroids, she still had a platelet count of only 30 x 10^9^/L platelets, which required the anticoagulation to be interrupted. It was then decided to readmit the patient to further investigate other causes of secondary thrombocytopenia and/or assess the need for second-line therapies such as splenectomy or rituximab.

**Figure 1:**
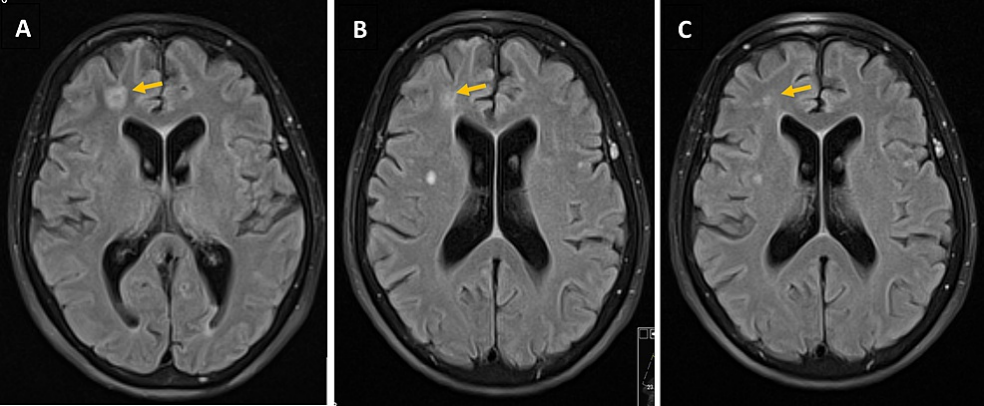
Evolution of brain infectious lesions on cerebral magnetic resonance. 1A: Lesion (arrow) on day 3 of treatment; 1B: Lesion (arrow) on day 47 of treatment; 1C: Lesion (arrow) on day 68 of treatment.

Upon admission, a few days later, she had a platelet count <10 x 10^9^/L, with only rare petechiae dispersed on the lower limbs. She had no other complaints, nor did she have other changes on physical examination. On that day, due to minor epistaxis, she was transfused with pooled platelets. The next morning, the patient complained of intense headache, with associated photophobia, with no improvement after paracetamol 1 g. Then she presented with a high fever (temperature >40º C) with poor response to antipyretics. She had no other symptoms suggesting an active site of infection. The cerebral CT showed no remarkable changes, and the laboratory results only showed leucocytosis with neutrophilia (displayed in Table 1). After blood and urine recollection for culture, she was empirically given piperacillin-tazobactam. Her state deteriorated rapidly with the loss of consciousness during that night, presenting a Glasgow Coma Scale (GCS) of 8 points, maintaining a high fever. The cerebral CT was repeated but showed no changes. A lumbar puncture was performed; however, the samples were clotted, invalidating the results. The antimicrobial was empirically adjusted for ceftriaxone, ampicillin, and acyclovir. On that same day, a coccobacillus was identified on the blood culture of the day before, which ultimately was confirmed to be *Listeria monocytogenes*. Consequently, only 36 hours after the onset of symptoms, the treatment was adjusted to ampicillin plus gentamicin. On the third day of treatment, a cerebral magnetic resonance (C-MRI) showed signs of supratentorial cerebritis with little pyogenic foci, but without a defined encapsulation (Figure 1A). Despite the CNS infection, it was decided to maintain prednisolone 80 mg/day, and it was necessary to administer immunoglobulin three times during the hospital stay for severe thrombocytopenia associated with sepsis. The patient rapidly recovered her consciousness, maintaining complaints of headache and photophobia but without any other symptoms or neurological deficits upon physical examination. Those symptoms disappeared over two weeks. With the improvement of the infection, the platelet counts and the inflammatory markers also improved. The subsequent blood cultures were negative, as early as 48 hours of the onset of ampicillin. She underwent seven days of gentamicin and ampicillin, after which we maintained ampicillin only for six more weeks. The radiological revaluation in week 7 still showed some infectious lesions, of smaller size but still with diffusion restriction and contrast enhancement, suggesting active infection (Figure 1B). We repeated the lumbar puncture, with cytology of the liquor showing only three cells, normal biochemical counts, negative culture, and negative search for *L. monocytogenes* DNA via polymerase chain reaction (PCR). At week 10 of treatment, the C-MRI showed smaller lesions and without contrast enhancement (Figure 1C), so ampicillin was stopped on day 73 of treatment.

**Table 1:** Summary of laboratory findings. CRP: C-reactive protein; LDH: Lactate dehydrogenase; CMV: Cytomegalovirus; EBV: Epstein-Barr virus, IgM: Immunoglobulin M.

Parameter	Value	Ref. Value
Hemoglobin	10.7 g/dL	12.0-16.0
Leucocytes	18.14 × 10^9^/L	4.0-11.0
Neutrophils	91.4%	53.8-69.8
Platelets	25 × 10^9^/L	150-400
LDH	382 U/L	<225
CRP	7.9 mg/L	<3.0
Creatinine	0.43 mg/dL	0.51-0.95
Urea	28 mg/dL	10-50
Leucocytes (urine)	11.4 g/L	<30.0
Erythrocytes (urine)	54.2/µL	<27.0
Nitrites (urine)	Negative	
Leptospira (urine)	Negative	
Leptospira (serum)	Negative	
Anti-CMV	IgM negative	
Anti-Parvovírus B19	IgM negative	
Anti-EBV	IgM negative	
Anti-Rickettsia conorii	IgM negative	
Blood cultures	Cocobacillus gram-positive identified as Listeria monocytogenes	

Concerning the thrombocytopenia, because the patient recurrently needed IV immunoglobulin (despite ongoing therapy with prednisolone 80 mg/day), and because she had a high risk for infection with immunosuppression, it was decided that it was not prudent to start her on rituximab at that stage, so she was started with eltrombopag, a thrombopoietin agonist. Initially, the response was weak, so she underwent a myelogram and bone marrow biopsy that showed no abnormal precursors, presenting a high count of eumorphic megakaryocytes. The dose of eltrombopag was uptitrated and two weeks later we obtained a sustained response, with an increase in platelet count, permitting a slow withdrawal of corticosteroids and the reintroduction of anticoagulation therapy, first with enoxaparin, later switched to apixaban. The patient was discharged after 75 days of hospitalization, with no symptoms and no neurological deficits, with a platelet count of 152 x 10^9^/L.

## Discussion

ITP is characterized by the destruction of platelets by antibodies against autoantigens present in them. It is one of the most common causes of thrombocytopenia in young adults and it can be divided into those which are truly immune and those which are secondary to other diseases, such as hepatitis C or neoplasms. Its incidence is 1 to 3 cases per 100,000, but its prevalence is four times larger since it is a rather benign condition. The disease usually manifests itself with minor hemorrhages, most of the time not requiring treatment, even when the platelet count is very low. However, some patients with higher hemorrhagic risk or in need of anticoagulation must have a platelet count over 50 x 10^9^/L, often needing treatment at some point. The first-line treatment is immunosuppression with prednisolone 1 mg/Kg/day which, after restoration and stabilization of platelet counts without bleeding, can be gradually tapered to the minimal effective dosage or preferably to suspension, according to the patient’s response. Immunosuppression carries along with relevant side effects, such as the increased risk for opportunistic infections [1].

*L. monocytogenes* is mostly a gram-positive bacillus; however, it can present itself as a coccobacillus. It can easily evade the immune system and be transmitted by the fecal-oral route. The incidence of a Listeria infection is 3-6 cases per million, but its prevalence has been increasing. The incubation time can be of 11 days but can go as long as 70 days. There are two types of infection: non-invasive, like febrile enteritis, and the invasive type that usually affects immunocompromised patients and pregnant women and their newborns. These bacteria have a predilection for the CNS, the reason why neurolisteriosis can account for as much as half the cases of invasive listeriosis, the remaining cases being usually isolated bacteremia or disease in pregnancy. Listeriosis has a high mortality rate of 20-30%, but neurolisteriosis can increase this rate to more than 50%, while survivors can have significant morbidity due to neurological sequelae, present in up to two-thirds of the patients. There are three types of neurolisteriosis: meningitis/meningoencephalitis, rhombencephalitis, and cerebritis, usually evolving to cerebral abscesses [2-9]. When comparing neurolisteriosis with more common bacterial meningitis, the first is more insidious with less prevalence of neck stiffness but more cases of altered consciousness and neurological sequelae. The analysis of cerebral spinal fluid is less informative, presenting normal glucose levels and with a low rate of isolating the pathogen in fluid cultures (only 11-41%). Listeria is more often identified in blood cultures (up to 60% of the neurolisteriosis) [3, 8-10]. A C-MRI should be performed to evaluate the lesions and properly assess the extension of the disease, as well as monitor treatment [3, 6, 11]. Ampicillin is the antimicrobial of choice, either isolated or combined with one week of gentamicin for synergy, but this data is controversial, with contradictory results in different studies. Given that the in vitro activity of gentamicin as a bactericidal is strong, we preferred the combination of antimicrobials and observed no relevant side effects. Cotrimoxazole could be an alternative, at least for the final part of the treatment, allowing for an oral route of administration. However, there are still not enough data to recommend it [3-5, 8, 9, 12, 13]. Corticosteroids have been used with beneficial results in common forms of bacterial meningitis. However, some studies tried this adjuvant treatment in neurolisteriosis, which showed either no statistically significant benefit or even increased morbimortality. We should state that this data is derived from observational studies only, most of which are retrospective. Therefore, corticosteroid adjuvant therapy cannot be recommended in neurolisteriosis [4, 5, 8-10, 12]. In our case, the maintenance of prednisolone 80 mg/day was required because of the ITP. Regarding the duration of the antimicrobial treatment, there is no certainty either. A minimum duration of 6-8 weeks of treatment is recommended for cerebritis with abscesses, so long as the lesions have improved or disappeared, when evaluated by C-MRI [3-5, 8, 9, 12, 13]. Therefore, we revaluated our patient with an MRI at week 8 of treatment and prolonged the treatment for a few more weeks until the lesions were no longer enhanced by the contrast.

## Conclusions

With this report, we want to stress out that neurolisteriosis can be a severe condition, with high morbimortality. The average delay time between the beginning of the symptoms and treatment can be as high as seven days, with an impact on prognosis. The fact that this patient was already admitted to a medical ward when she first presented symptoms assured a prompt diagnosis and the beginning of effective treatment in less than 36 hours. This certainly contributed to the astounding clinical improvement, with a resolution of neurological symptoms and the lack of sequelae, despite the need for maintaining immunosuppressant therapy.
